# Genetic Vaccination against Experimental Infection with Myotropic Parasite Strains of *Trypanosoma cruzi*

**DOI:** 10.1155/2014/605023

**Published:** 2014-06-26

**Authors:** Adriano Fernando Araújo, Gabriel de Oliveira, Juliana Fraga Vasconcelos, Jonatan Ersching, Mariana Ribeiro Dominguez, José Ronnie Vasconcelos, Alexandre Vieira Machado, Ricardo Tostes Gazzinelli, Oscar Bruna-Romero, Milena Botelho Soares, Mauricio Martins Rodrigues

**Affiliations:** ^1^Centro de Terapia Celular e Molecular (CTCMol), Escola Paulista de Medicina, UNIFESP, Rua Mirassol, 207 04044-010 São Paulo, SP, Brazil; ^2^Departmento de Microbiologia, Imunologia e Parasitologia, Escola Paulista de Medicina, Universidade Federal de São Paulo, Rua Mirassol, 207 04044-010 São Paulo, SP, Brazil; ^3^Laboratório Biologia Celular, Instituto Oswaldo Cruz (FIOCRUZ), Avenida Brasil, 4365—Manguinhos, 21040-360 Rio de Janeiro, RJ, Brazil; ^4^Centro de Pesquisas Gonçalo Moniz, FIOCRUZ, Rua Waldemar Falcão, 121, 40296-710 Salvador, BA, Brazil; ^5^Hospital São Rafael, Avenida São Rafael 2152, São Marcos, 41253-190 Salvador, BA, Brazil; ^6^Departamento de Biociências, Instituto de Saúde e Sociedade, UNIFESP, Campus Baixada Santista, 11015-020 Santos, SP, Brazil; ^7^Centro de Pesquisas René Rachou, FIOCRUZ, Avenida Augusto de Lima 1.715, Barro Preto, 30190-002 Belo Horizonte, MG, Brazil; ^8^Departamento de Bioquímica e Imunologia, Instituto de Ciências Biológicas, Universidade Federal de Minas Gerais, Avenida Presidente Antônio Carlos, 6627, Pampulha, 31270-901 Belo Horizonte, MG, Brazil; ^9^Division of Infectious Disease and Immunology, Department of Medicine, University of Massachusetts Medical School, 55 Lake Avenue North, Worcester, MA 01655, USA; ^10^Departamento de Microbiologia, Imunologia e Parasitologia, Universidade Federal de Santa Catarina, Campus Universitário da Trindade, 88040-900 Florianopolis, SC, Brazil

## Abstract

In earlier studies, we reported that a heterologous prime-boost regimen using recombinant plasmid DNA followed by replication-defective adenovirus vector, both containing *Trypanosoma cruzi* genes encoding *trans*-sialidase (TS) and amastigote surface protein (ASP) 2, provided protective immunity against experimental infection with a reticulotropic strain of this human protozoan parasite. Herein, we tested the outcome of genetic vaccination of F1 (CB10XBALB/c) mice challenged with myotropic parasite strains (Brazil and Colombian). Initially, we determined that the coadministration during priming of a DNA plasmid containing the murine IL-12 gene improved the immune response and was essential for protective immunity elicited by the heterologous prime-boost regimen in susceptible male mice against acute lethal infections with these parasites. The prophylactic or therapeutic vaccination of resistant female mice led to a drastic reduction in the number of inflammatory infiltrates in cardiac and skeletal muscles during the chronic phase of infection with either strain. Analysis of the electrocardiographic parameters showed that prophylactic vaccination reduced the frequencies of sinus arrhythmia and atrioventricular block. Our results confirmed that prophylactic vaccination using the TS and ASP-2 genes benefits the host against acute and chronic pathologies caused by *T. cruzi* and should be further evaluated for the development of a veterinary or human vaccine against Chagas disease.

## 1. Introduction

Chagas disease is an acute and chronic illness caused by* Trypanosoma cruzi*, an obligatory intracellular protozoan parasite that is endemic in the Americas. The disease currently affects millions of people who are chronically infected. Thousands of new cases are also estimated to occur every year [1, 2]. Prophylactic measures aimed at eliminating transmission by the vector (kissing bugs) have been very successful in many countries [3]. Considering their success and the highly neglected state of Chagas disease research, vaccine development has often been considered a difficult and expensive strategy for disease control and eradication.

Despite this general consideration, recent theoretical studies have shown a divergent perspective on the problem, indicating that the development of a vaccine against Chagas disease would be cost effective even if the efficacy is not high and the transmission is low [4, 5]. In moving toward the development of vaccines against Chagas disease, several laboratories have described a number of antigens and delivery systems capable of providing some degree of protective immunity against experimental infections. These systems include genetically attenuated parasites, recombinant protein in adjuvant systems, and genetically modified bacteria or viral vectors (reviewed in [6–12]). The antigens pursued for recombinant vaccines include* T. cruzi trans*-sialidase, cruzain (a cysteine proteinase), and the amastigote surface proteins 2, 3, 4, TcG1, TcG2, TcG4, TSA-1, and Tc24 (reviewed in [6–12]).

Our group in particular has been working for many years on the development of genetic vaccines against* T. cruzi* infection. We initially used the gene encoding the catalytic domain of the parasite* trans*-sialidase (TS, [13, 14]). More recently, we have described studies using a heterologous prime-boost regimen consisting of priming with plasmid DNA followed by a booster injection with replication-defective recombinant human adenovirus type 5. Both the prime and the boost contained the gene encoding the amastigote surface protein (ASP)-2 antigen of* T. cruzi.* This regimen successfully vaccinated highly susceptible A/Sn and resistant C57BL/6 mice against infection with the reticulotropic Y strain of* T. cruzi* [15–18]. Protective immunity was mediated by long-lived CD4^+^ and CD8^+^ T Effector or effector memory cells [15, 19, 20].

*T. cruzi* infection in mammalian hosts leads to diverse clinical manifestations. Among the most relevant factors influencing this diversity is the existence of biologically different parasite strains.* T. cruzi* intraspecific nomenclature was established in 2009, and the isolates and strains are assigned to one of six genetic groups or discrete typing units, named TcI to TcVI [21]. Among domestic transmission cycles, TcI occurs predominantly in northern South America, while TcII, TcV, and TcVI are more often observed in the Southern Cone countries [22]. Considering this genetic variability, it is important that studies of vaccination or chemotherapy are conducted in different experimental models using distinct parasite strains. Because our previous studies of vaccination have been performed with the reticulotropic Y strain (TcII), it was our intention to extend these studies using experimental infections with two myotropic strains of the parasites belonging to TcI (Colombian) and TcII (Brazil).

## 2. Materials and Methods

### 2.1. Ethics Statement

This study was carried out in strict accordance with the recommendations in the Guide for the Care and Use of Laboratory Animals of the Brazilian National Council of Animal Experimentation (http://www.cobea.org.br/). The protocol was approved by the Committee on the Ethics of Animal Experiments of the Institutional Animal Care and Use Committee at the Federal University of Sao Paulo (Id # CEP 0426/09).

### 2.2. Mice and Parasites

Female or male 5- to 8-week-old F1 (CB10XBALB/c) mice were purchased from CEDEME (Federal University of São Paulo). Bloodstream trypomastigotes of the Colombian or Brazil strain of* T. cruzi* were obtained from mice infected 21–28 days earlier. The concentration of parasites was estimated and adjusted to 10^4^ parasites/mL. Each mouse was inoculated with 10^3^ trypomastigotes diluted in 0.1 mL PBS and administrated subcutaneously (s.c.) in the base of the tail.

### 2.3. Peptides

Synthetic peptides (Genscript, Piscataway, New Jersey) were higher than 90% pure. The immunodominant epitopes of ASP-2 or TS were represented by AA VNHRFTLV or IYNVGQSVI, respectively.

### 2.4. Genetic Vaccination

The plasmid (pWRG3169) containing coding sequences for the p35 and p40 subunits of murine (m) IL-12 was generated as earlier described [23]. Plasmids p154/13 (TS gene) or pIgSPclone9 (ASP-2 gene) were generated, grown, and purified as described earlier [13, 24]. Human replication deficient adenovirus type 5 expressing the ASP-2 gene (AdASP-2) or TS gene (AdTS) was generated and produced as described earlier [25] Control mice were immunized with pcDNA3 and human replication deficient adenovirus type 5 expressing *β*-galactosidase (Ad*β*-gal). F1 (CB10XBALB/c) mice were immunized i.m. in each* tibialis anterioris* muscle with plasmid DNA. For priming, control mice received plasmid pcDNA3 (300 *μ*g), a second group was immunized with a mixture of pIgSPCl9 and p154/13 (200 *μ*g), and the third, a mixture of pIL12, pIgSPCl9, and p154/13 (300 *μ*g). Twenty-one days later, these mice received in these same spots 100 *μ*L of viral suspension containing a total of 2 × 10^8^ plaque forming units (pfu) of rec. adenovirus. For boosting, control mice received adenovirus Ad*β*gal; other mouse groups were immunized with a mixture of rec. adenovirus AdASP-2 and Ad-TS. Immunological assays were performed 14 days after viral inoculation.

Therapeutic vaccination was performed as follows. F1 (CB10XBALB/c) female mice were challenged s.c. with 1,000 trypomastigotes of the indicated parasite strain. Thirty days later, control mice received a mixture of plasmid pcDNA3 and of pIL12 (300 *μ*g) and second mice group was immunized with a mixture of pIL12pIgSPCl9 and p154/13 (300 *μ*g). Twenty days later, these mice received in these same spots 100 *μ*L of viral suspension containing a total of 2 × 10^8^ pfu of rec. adenovirus. For boosting, control mice received adenovirus Ad*β*gal and the other mice were immunized with a mixture of rec. adenovirus AdASP-2 and Ad-TS.

### 2.5. Immunological Assays

For the surface intracellular expression of cytokines (IFN*γ* and TNF, ICS) or translocation of CD107a to the membrane, splenocytes collected from immune mice were treated with ACK buffer. ICS and surface mobilization of CD107a were evaluated after* in vitro* culture of splenocytes in the presence or absence of the antigenic stimulus. Cells were washed 3 times in plain RPMI and resuspended in cell culture medium consisting of RPMI 1640 medium, pH 7.4, supplemented with 10 mM Hepes, 0.2% sodium bicarbonate, 59 mg/L of penicillin, 133 mg/L of streptomycin, and 10% Hyclone fetal bovine sera (Hyclone, Logan, Utah). The viability of the cells was evaluated using 0.2% trypan blue exclusion dye to discriminate between live and dead cells. Cell concentration was adjusted to 5 × 10^6^ cells/mL in cell culture medium containing FITC-labeled anti-CD107a (2 *μ*g/mL), anti-CD28 (2 *μ*g/mL), BdGolgiPlug, and monensin (5 *μ*g/mL). In part of the cultures, a final concentration of 10 *μ*M of the VNHRFTLV or IYNVGQSVI peptides was added. The cells were cultivated in flat-bottom 96-well plates (Corning) in a final volume of 200 *μ*L in duplicate, at 37°C in a humid environment containing 5% CO_2_. After 12 h incubation, cells were stained for surface markers with PerCP-labeled anti-CD8, on ice for 20 min. To detect IFN*γ* and TNF by intracellular staining, cells were then washed twice in buffer containing PBS, 0.5% BSA, and 2 mM EDTA, fixed and permeabilized with BD perm/wash buffer. After being washed twice with BD perm/wash buffer, cells were stained for intracellular markers using APC-labeled anti-IFN*γ* (Clone XMG1.2) and PE-labeled anti-TNF (clone MP6-XT22). Finally, cells were washed twice with BD perm/wash buffer and fixed in 1% PBS-paraformaldehyde. At least 300,000 cells were acquired on a BD FacsCanto flow cytometer and then analyzed with FlowJo.

### 2.6. Electrocardiogram (ECG)

Mice were i.p. tranquilized with diazepan (20 mg/kg) and transducers were carefully placed under the skin in accordance with chosen preferential derivation (DII). Traces were recorded using a digital system (Power Lab 2/20) connected to a bioamplifier in 2 mV for 1 s (PanLab Instruments). Filters were standardized between 0.1 and 100 Hz and traces were analyzed using the Scope software for Windows V3.6.10 (PanLab Instruments). We measured heart rate (beats per minute—bpm), duration of the PR, QRS, and QT intervals, and P wave in ms (millisecond) on 222 dpi. The relationship between the QT interval and RR interval was individually assessed to obtain physiologically relevant values for the heart rate-corrected QT interval (QTc) through Bazzet's formula.

### 2.7. Morphometric Analyses

Analyses were performed essentially as described in [26]. Briefly, heart sections were analyzed by light microscopy after paraffin embedding, followed by standard hematoxylin and eosin staining. Inflammatory cells infiltrating heart tissue were counted using a digital morphometric evaluation system. Images were digitized using a color digital video camera (CoolSnap, Photometrics, Montreal, QC, Canada) adapted to a BX41 microscope (Olympus, Tokyo, Japan). Morphometric analyses were performed using the software Image-Pro Plus v.7.0 (Media Cybernetics*¸* San Diego, CA, USA). The inflammatory cells were counted in 10 fields (×400 view)/heart. All of the analyses were performed in a blinded fashion.

### 2.8. Statistical Analysis

For the purpose of comparing the different mouse groups, we used the statistical analyses suggested by [27]. The values were compared using One Way ANOVA followed by Tukey's HSD tests or Fisher exact probability test (http://faculty.vassar.edu/lowry/VassarStats.html/). The Logrank test was used to compare mouse survival rates after challenge with* T. cruzi* (http://bioinf.wehi.edu.au/software/russell/logrank/). The differences were considered significant when the *P* value was <0.05.

## 3. Results

### 3.1. Addition of pIL-12 during Priming Improved the Immune Response Mediated by CD8^+^ T Cells and Increased Protective Immunity against Acute Infection

A previous study has reported that the coadministration of plasmid DNA expressing IL-12 during priming improves the efficacy of a heterologous prime-boost vaccination regimen, as estimated by protective immunity against SIV infection [28]. We, therefore, decided to determine whether this strategy could also improve the immune response and protective immunity against* T. cruzi* infection in the mouse model. Mice were immunized with the following: (i) pcDNA3 followed by a booster injection of Ad*β*gal (G1, control); (ii) p154/13 and pIgSPCl9 followed by a booster injection of AdASP-2 and AdTS (G2); (iii) pIL-12, p154/13 and pIgSPCl9 followed by a booster injection of AdASP-2 and AdTS (G3).

**Figure 1:**
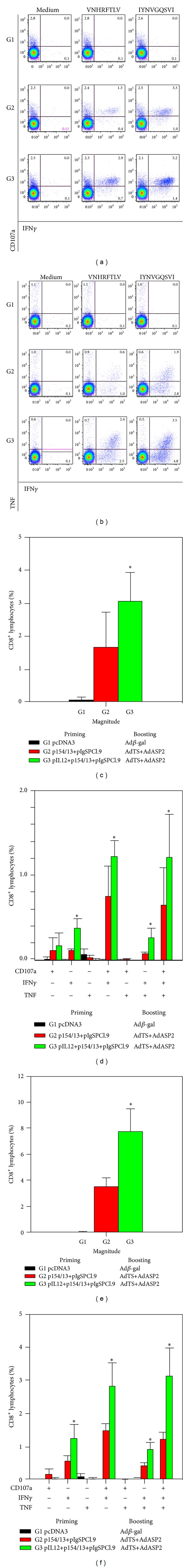
Frequencies of specific cytokine-secreting splenic CD8^+^ T cells in mice vaccinated with the heterologous prime-boost vaccination regimen. F1 (CB10XBALB/c) mice were immunized i.m. in each* tibialis anterior* muscle with plasmid DNA. For priming, G1 received control plasmid pcDNA3 (300 *μ*g), G2 was immunized with a mixture of pIgSPCl9 and p154/13 (200 *μ*g), and G3 received a mixture of pIL-12, pIgSPCl9, and p154/13 (300 *μ*g). Twenty-one days later, these mice received, in these same spots, 100 *μ*L of a viral suspension containing a total of 2 × 10^8^ pfu of rec. adenovirus. For boosting, G1 received control adenovirus Ad*β*gal, and G2 and G3 were immunized with a mixture of rec. adenovirus AdASP-2 and Ad-TS. Immunological assays were performed 14 days after viral inoculation. Spleen cells were restimulated* in vitro* in the presence or absence of the peptides VNHRFTLV or IYNVGQSVI, anti-CD107a, anti-CD28, BdGolgiPlug, and monensin. After 12 h, the cells were stained with anti-CD8, anti-IFN*γ*, and anti-TNF. (a) The histograms show FACS analysis of CD8+ cells. The numbers represent the frequencies of cells stained for CD107a and/or IFN*γ*. The results are a representative of 4 mice per group (Median). (b) Frequencies of CD8+ cells stained for IFN*γ* and/or TNF. The results are a representative of 4 mice per group (Median). (c) Total frequencies of CD8+ cells specific for the peptide VNHRFTLV and stained for CD107a or IFN*γ* or TNF. The results are representative of 4 mice per group (Mean ± SD). (d) Frequencies of CD8+ cells specific for the peptide VNHRFTLV and stained for each marker (CD107a, IFN*γ*, or TNF). The results are representative of 4 mice per group (Mean ± SD). (e) Same as in (c) for CD8+ cells specific for the peptide IYNVGQSVI. The results are representative of 4 mice per group (Mean ± SD). (f) Same as in (d) for CD8+ cells specific for the peptide IYNVGQSVI. All three groups were compared statistically by one-way ANOVA followed by Tukey HSD (c–e). The values of G2 and G3 mice were always higher than those of G1 mice (*P* < 0.01 in all cases). Asterisks denote that the values of G3 mice were higher than those of G2 mice (*P* < 0.05). The results are representative of three independent experiments.

To compare the different mouse groups, we stained CD8^+^ spleen cells following* in vitro* peptide stimulation for the surface mobilization of CD107a, a marker for T cell degranulation, and intracellular effector cytokines (IFN*γ* and TNF, ICS).  Figure 1(a) depicts examples of the frequency estimates of peptide-specific CD8^+^ spleen cells that mobilized CD107a to their surface and expressed IFN*γ*. The frequencies of CD8^+^ cells that mobilized CD107a to the cell surface and expressed IFN*γ* were higher in G3. Control mice (G1) had a negligible number of specific CD8^+^CD107a^+^IFN*γ* ^+^ cells. These frequencies were dependent on the presence of peptide in culture because the frequency of CD8^+^CD107a^+^IFN*γ* ^+^ cells was very small in the absence of peptide in all groups.

We made a similar observation when we estimated the frequencies of splenic peptide-specific CD8 cells that expressed IFN*γ* or IFN*γ* and TNF. These frequencies were higher in G3 mice (Figure 1(b)). As described above, control mice (G1) had a negligible number of specific CD8^+^IFN*γ* ^+^ or CD8^+^IFN*γ* ^+^TNF^+^ cells. The numerical differences and the statistical significances are depicted in Figures 1(c)–1(f). As shown in Figures 1(d) and 1(f), following peptide stimulation* in vitro*, most of the CD8^+^ cells were either CD107a^+^IFN*γ* ^+^TNF^+^ or CD107a^+^IFN*γ* ^+^.

**Figure 2:**
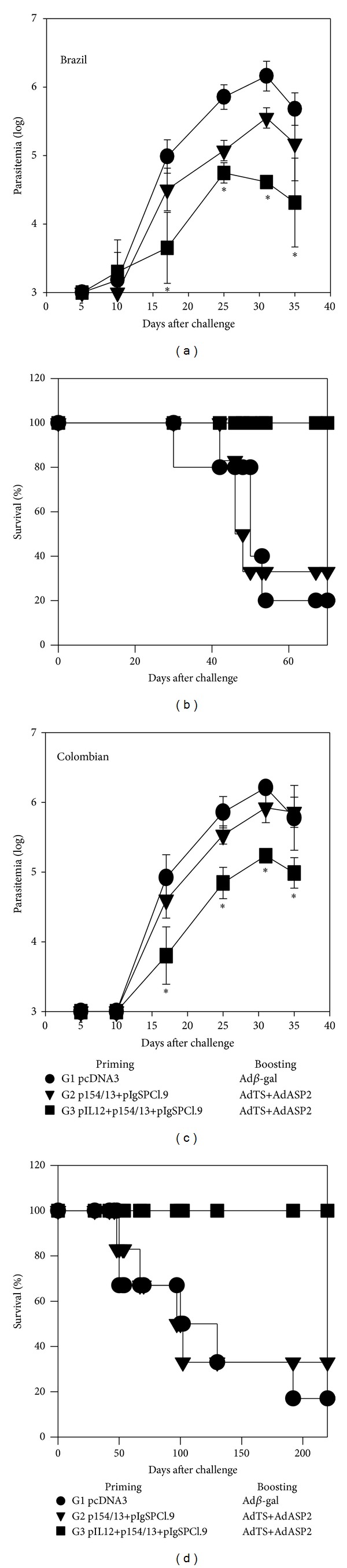
The protective immunity elicited by heterologous prime-boost vaccination in susceptible mice. F1 (CB10XBALB/c) male mice were immunized i.m. exactly as described in the legend of Figure 1. Fourteen days later, mice were challenged s.c. with 1,000 trypomastigotes of the indicated parasite strain. (a) Mean parasitemia ± SD of each mouse group (*n* = 6) challenged with parasites of the Brazil strain. The values of G2 and G3 mice at days 21, 25 and 35 were lower than those of G1 mice (*P* < 0.01 in all cases, one-way ANOVA, Tukey HSD). Asterisks denote that the values of G3 mice were lower than those of G2 mice (*P* < 0.05); (b) The Kaplan-Meier survival curves of the different groups were compared, and the results showed that G3 survived significantly longer (*P* < 0.01) than the two other groups; (c) Mean parasitemia ± SD of each mouse group (*n* = 6) challenged with parasites of the Colombian strain. The values of G2 and G3 mice at days 25 and 31 were lower than those of G1 mice (*P* < 0.01 in all cases). Asterisks denote that the values of G3 mice were lower than those of the G2 mice (*P* < 0.05); (d) The Kaplan-Meier survival curves of the different groups were compared, and the results showed that G3 survived significantly longer (*P* < 0.01) than the two other groups. The results were obtained from one of two independent experiments.

To determine whether this improvement in the frequencies of specific CD8^+^ T cells would impact protective immunity, we vaccinated susceptible male mice and challenged them with trypomastigotes of either the Brazil or Colombian strain. As shown in Figures 2(a) and 2(c), mice vaccinated with the TS and ASP-2 genes (G2 and G3) presented a statistically significant reduction in peak parasitemia. A comparison between these two groups also showed that the parasitemia in G3 was significantly lower than that in G2. Not only did mice from G3 display lower parasitemia, but vaccination also significantly reduced mouse mortality (Figures 2(b) and 2(d)). While all G3 mice survived the experimental challenge, most control (G1) and G2 mice died after challenge with the Brazil or Colombian parasite strain. Based on these results, we concluded that the use of pIL-12 for priming significantly improved the immune response and the protective immunity against acute infection with the myotropic Brazil and Colombian strains.

### 3.2. Impact of Genetic Vaccination against Chronic Infection

To evaluate the impact of genetic vaccination on the chronic symptoms of the experimental infection, resistant female mice were treated according to the following protocols: (i) naïve (G1); (ii) pIL-12 and pcDNA3 followed by a booster injection of Ad*β*gal (G2, control); (iii) pIL-12, p154/13 and pIgSPCl9 followed by a booster injection of AdASP-2 and AdTS (G3).

**Figure 3:**
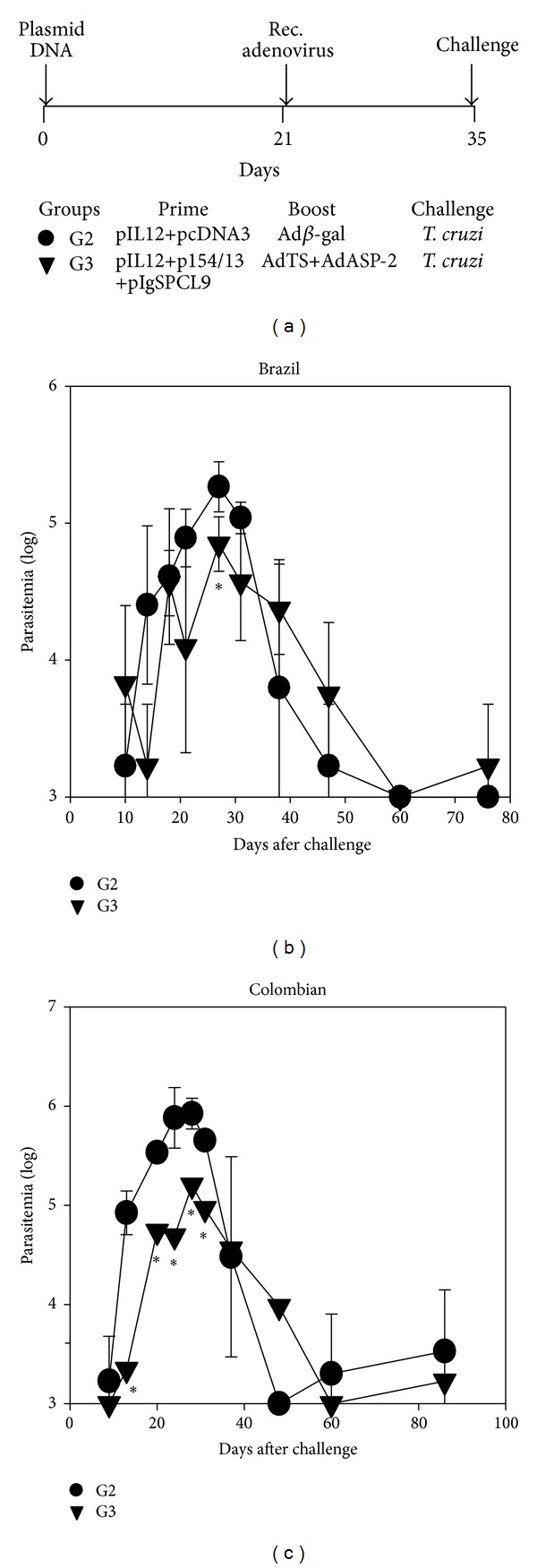
The protective immunity elicited by heterologous prime-boost vaccination in resistant mice. (a) F1 (CB10XBALB/c) female mice were immunized i.m. as depicted. For priming, G2 mice received a mixture of control plasmid pcDNA3 and of pIL-12 (300 *μ*g), and G3 mice were immunized with a mixture of pIL-12, pIgSPCl9 and p154/13 (300 *μ*g). Twenty-one days later, these mice received, in these same spots, 100 *μ*L of a viral suspension containing a total of 2 × 10^8^ pfu of rec. adenovirus. For boosting, G2 mice received control adenovirus Ad*β*gal, and G3 mice were immunized with a mixture of rec. adenovirus AdASP-2 and Ad-TS. Fourteen days later, mice were challenged s.c. with 1,000 trypomastigotes of the indicated parasite strain. (b) Mean parasitemia ± SD of each mouse group (*n* = 6) challenged with parasites of the Brazil strain. Asterisk denotes that at day 37, the values of G3 mice were lower than those of G2 mice (*P* < 0.05, one-way ANOVA, Tukey HSD); (c) Mean parasitemia ± SD of each mouse group (*n* = 6) challenged with parasites of the Colombian strain. Asterisks denote that at days 13 to 31, the values of G3 mice were lower than those of G2 mice (*P* < 0.05).

G2 and G3 mice were subsequently challenged with parasites of either the Brazil or the Colombian strain, as depicted in Figure 3(a). We estimated the level of parasitemia for each mouse group. G3 mice presented a lower peak parasitemia at day 27 (*P* = 0.02) after infection with parasites of the Brazil strain (Figure 3(b)). In the case of mice challenged with parasites of the Colombian strain, there was a statistically significant reduction in the levels of parasitemia at days 13, 20, 24, 28, and 31 (*P* < 0.05 in all cases, Figure 3(c)).

Individual ECGs were evaluated at days 80, 150, and 240 following infection. As shown in Table 1, the primary abnormalities we detected were sinus arrhythmia and atrioventricular block. Significantly lower frequencies of sinus arrhythmia were observed in G3 mice when compared with G2 mice following infection with the Brazil or Colombian strains. These differences were observed at later days (150 or 240 days) following challenge. Similarly, significantly lower frequencies of atrioventricular block were observed in G3 mice when compared with G2 mice following infection with parasites of the Brazil strain. In the case of mice challenged with parasites of the Colombian strain, the differences were smaller and not statistically significant.

**Figure 4:**
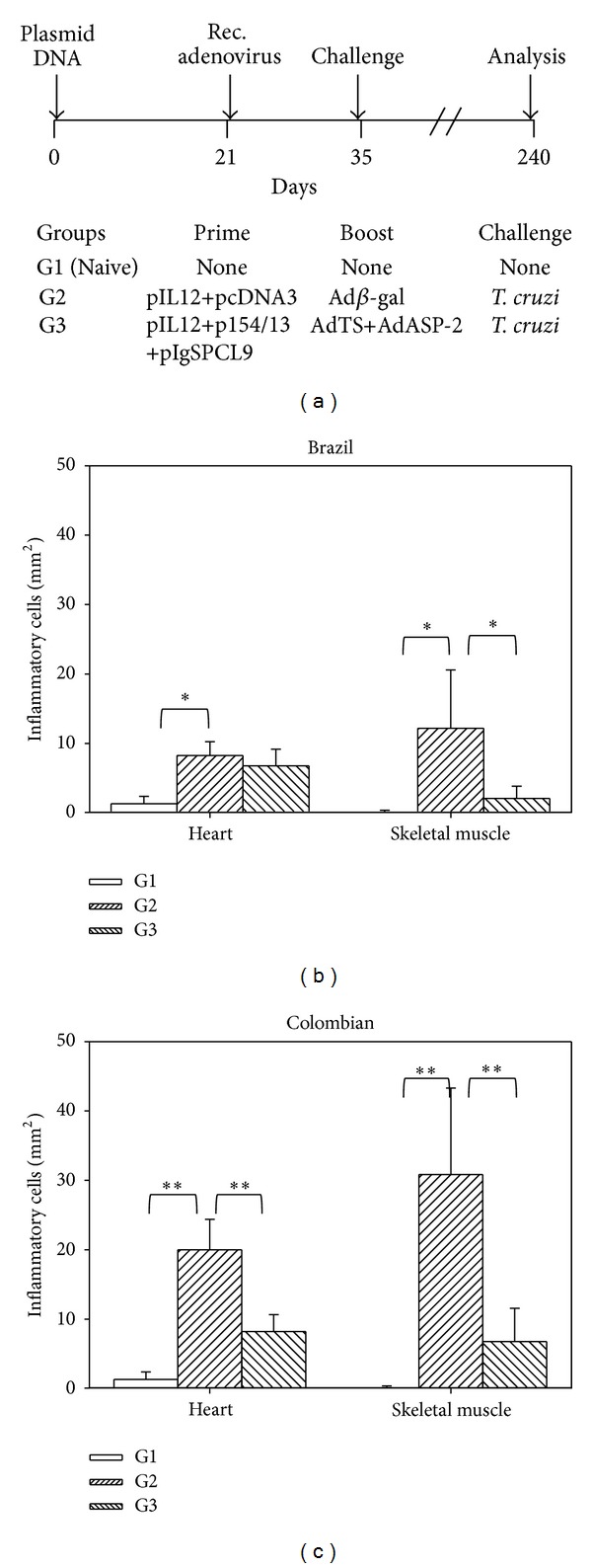
Frequencies of inflammatory cells in the hearts and skeletal muscles of resistant mice vaccinated by the heterologous prime-boost regimen. (a) F1 (CB10XBALB/c) female mice were immunized i.m., as depicted. Details on the dose are shown in Figure 3(a). (b) At 240 days, heart and skeletal muscle sections of mice challenged with the Brazil strain were stained with hematoxylin and eosin, and the number of inflammatory cells was quantified. Bars represent the mean of 6 mice/group ± SD. Asterisks denote that there was a significant difference between the indicated groups (*P < 0.05 or **P < 0.01). (c) The same as above, except that the mice were challenged with parasites of the Colombian strain. Bars represent the mean of 5 mice/group ± SD.

At day 240 after challenge, the mice were euthanized, and morphometric analyses of the heart and skeletal muscle were performed. The number of inflammatory cells within the skeletal muscle, but not the heart, of G3 mice challenged with parasites of the Brazil strain was significantly lower (Figure 4(b)). In G3 mice challenged with parasites of the Colombian strain, we found significantly lower numbers of inflammatory cells within the skeletal muscle and heart when compared to G2 mice (Figure 4(c)).

**Figure 5:**
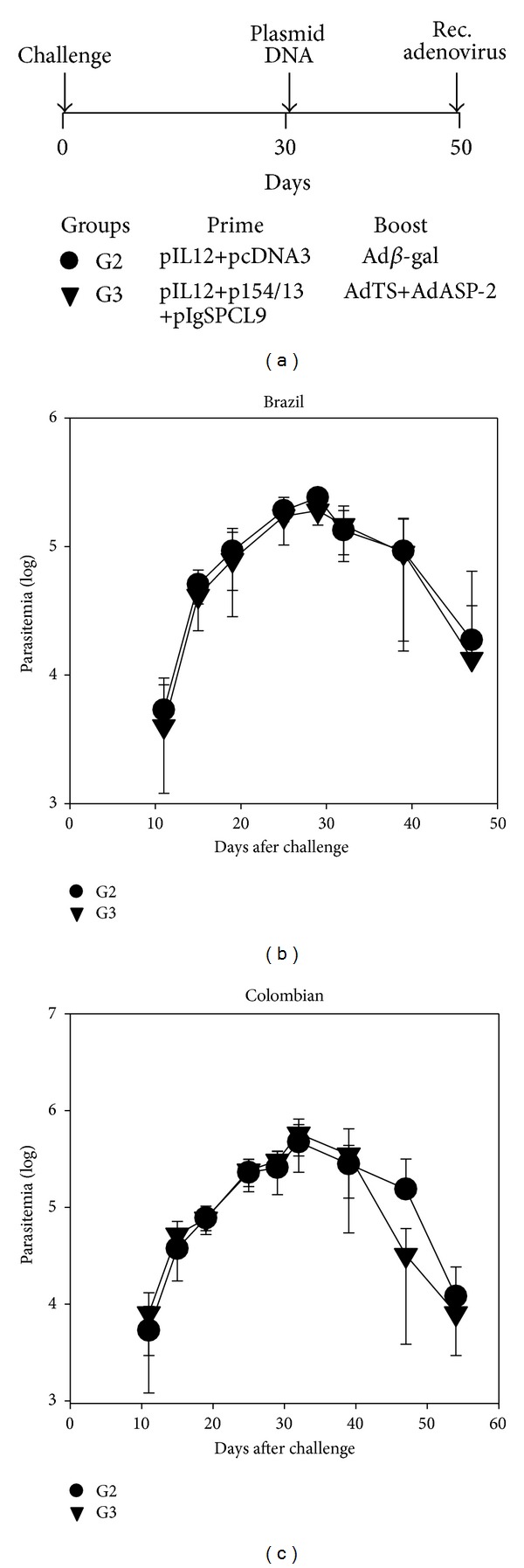
Impact of therapeutic vaccination in the immunity of resistant mice. (a) F1 (CB10XBALB/c) female mice were challenged s.c. with 1,000 trypomastigotes of the indicated parasite strain. Thirty days later, G2 mice received a mixture of control plasmid pcDNA3 and of pIL-12 (300 *μ*g), and G3 mice were immunized with a mixture of pIL-12, pIgSPCl9, and p154/13 (300 *μ*g). Twenty days later, these mice received, in these same spots, 100 *μ*L of a viral suspension containing a total of 2 × 10^8^ pfu of rec. adenovirus. For boosting, G2 mice received control adenovirus Ad*β*gal, and G3 mice were immunized with a mixture of rec. adenovirus AdASP-2 and Ad-TS. (b) Mean parasitemia ± SD of each mouse group (*n* = 6) challenged with parasites of the Brazil strain. (c) Mean parasitemia ± SD of each mouse group (*n* = 6) challenged with parasites of the Colombian strain.

**Figure 6:**
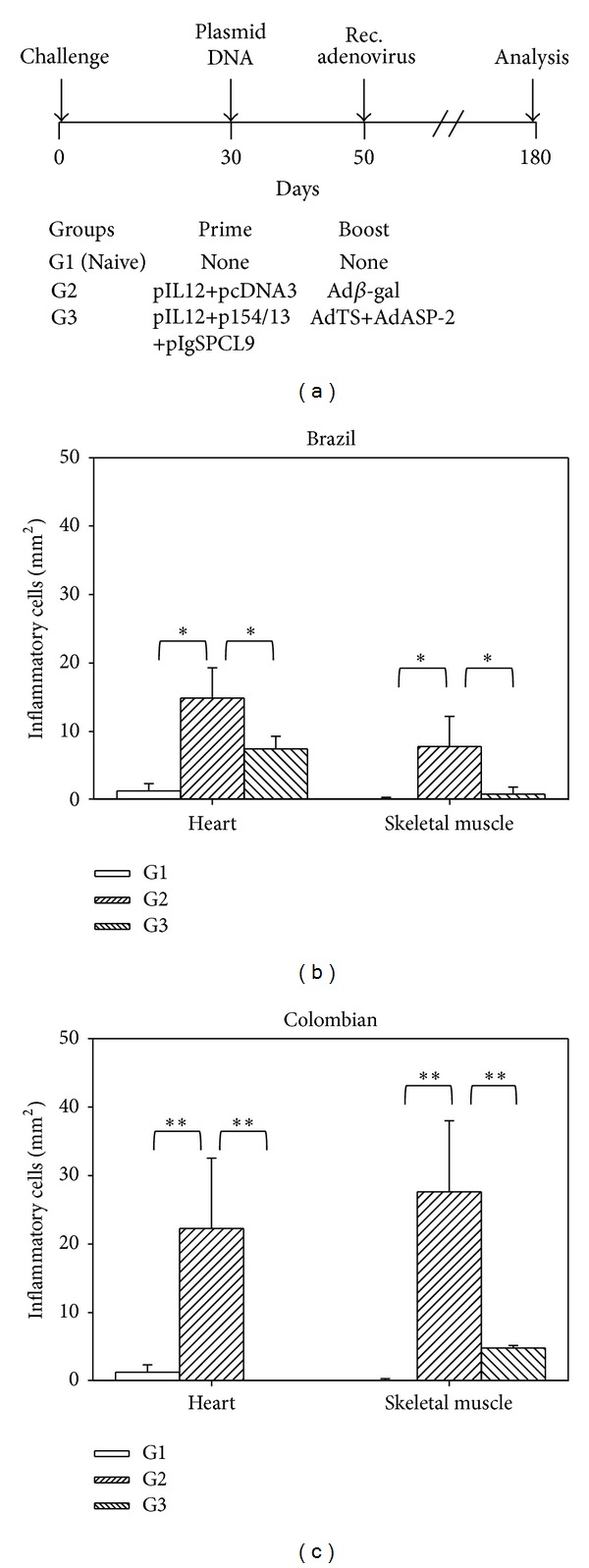
Frequencies of inflammatory cells in the hearts and skeletal muscles of resistant mice treated by the heterologous prime-boost regimen. (a) F1 (CB10XBALB/c) female mice were immunized i.m., as depicted. Details on the dose are shown Figure 5(a). (b) At 180 days, heart and skeletal muscle sections of mice challenged with the Brazil strain were stained with hematoxylin and eosin, and the number of inflammatory cells was quantified. Bars represent the mean of 6 mice/group ± SD. Asterisks denote that there was a significant difference between the indicated groups (*P < 0.05 or **P < 0.01). (c) The same as above, except that the mice were challenged with parasites of the Colombian strain.

We then evaluated the impact of therapeutic genetic vaccination on the chronic symptoms of experimental infection. Resistant female mice were infected and then treated according to the protocols described in Figures 5(a) and 6(a). Mice received the following treatments: (i) pIL-12 and pcDNA3 followed by a booster injection of Ad*β*gal (G2, control); (ii) pIL-12, p154/13 and pIgSPCl9 followed by a booster injection of AdASP-2 and AdTS (G3).

The estimation of parasitemia until day 50 did not reveal any difference between G2 or G3 mice infected with parasites of the Brazil or Colombian strains (Figures 5(b) and 5(c), resp.). We concluded that the plasmid administration at day 30 after challenge did not elicit immunity that could reduce the ongoing parasitemia. Individual ECGs were evaluated at days 90, 120, 150, and 180 following infection. As shown in Table 2, we detected differences in the frequencies of sinus arrhythmia, but not atrioventricular block, in G3 mice when compared with G2 mice following infection with the Brazil strain of* T. cruzi*. However, this difference was observed only on days 90 and 120. Later (150 and 180 days), G3 mice also developed sinus arrhythmia. G3 mice infected with parasites of the Colombian strain had significantly lower frequencies of sinus arrhythmia at days 120 and 180, when G2 mice developed a significant number of events. However, the occurrence of atrioventricular block in G3 mice was similar to that in G2. At day 180 after challenge, the mice were euthanized, and morphometric analyses of the heart and skeletal muscle were performed. The numbers of inflammatory cells within the skeletal muscle and heart of G3 mice challenged with parasites of the Brazil or Colombian strains were significantly lower than the frequencies in the tissues of G2 mice (Figures 6(b) and 6(c), resp.).

## 4. Discussion

The present study evaluated the outcome of experimental infection with two myotropic* T. cruzi* strains in F1 (CB10XBALB/c) mice genetically vaccinated with TS and ASP-2 following a heterologous DNA prime-adenovirus boost regimen. Prophylactic vaccination reduced the acute phase symptoms, as estimated by parasitemia and mortality. In addition, ECG analysis demonstrated that prophylactic vaccination also reduced some of the chronic phase signs. These results essentially confirm and extend our previous observations in mice challenged with a reticulotropic* T. cruzi* strain [15–20].

In contrast to prophylactic vaccination, the therapeutic use of our vaccine had a much lower impact on the chronic phase signs evaluated by ECG. The treatment reduced sinus arrhythmia earlier in mice challenged with the Brazil strain. Additionally, most mice challenged with the Colombian strain never developed episodes of sinus arrhythmia. Despite this observation, no differences were detected in the frequencies of episodes of atrioventricular block. It is important to highlight that these ECG alterations occurred in mice with a reduced number of inflammatory cells in their heart. This dichotomy most likely reflects the successful control by vaccination of the inflammatory reactions later during infection, when the mice were euthanized. However, the damage to the heart had been established earlier and could no longer be reversed.

The reason why the T cells elicited by therapeutic genetic vaccination are not capable of reducing the symptoms is unknown at present. One possibility is that following infection, specific T cells are already committed to express the death receptor CD95, as we have recently described [29]. Once these cells express CD95, their viability is impaired, and they can no longer expand properly. It will be important to test this possibility because it has implications for the use of T cell vaccines for therapeutic purposes during this or other chronic diseases [30–36].

The protective immune mechanisms mediating the effects of our new vaccination regimen were not addressed in the present study. However, it is plausible that CD4^+^ and CD8^+^ T cells participate in this immune response, as we have previously described [15]. Corroborating this hypothesis is the fact that the addition of pIL-12 during priming led to a significant increase in the frequencies of multifunctional CD8^+^ T cells and a parallel increase in the protective immunity against acute infection.

In conclusion, this study reinforces and extends our previous work by demonstrating the improvement in acute and chronic symptoms after prophylactic genetic vaccination against* T. cruzi* infection using a vector expressing the TS and ASP-2 antigens.

## Figures and Tables

**Table 1:** Summary of the ECG records.

Days after challenge	Strain	Sinus arrhythmia mouse groups			*P* values		
		G1	G2	G3	G1 × G2	G1 × G3	G2 × G3
80	Brazil	0/6	0/6	0/6	NS	NS	NS
150	Brazil	0/6	19/6	0/6	*0.001 *	NS	*0.001 *
240	Brazil	0/6	20/6	3/6	*0.001 *	NS	*0.026 *

80	Colombian	0/6	0/6	0/5	NS	NS	NS
150	Colombian	0/6	1/6	1/5	NS	NS	NS
240	Colombian	0/6	9/6	1/5	*0.017 *	NS	*0.093 *

Days after challenge	Strain	Atrioventricular block			*P* values		
		G1	G2	G3	G1 × G2	G1 × G3	G2 × G3

80	Brazil	0/6	0/6	0/6	NS	NS	NS
150	Brazil	0/6	4/6	2/6	*0.03 *	NS	NS
240	Brazil	0/6	59/6	7/6	*0.001 *	*0.004 *	*0.003 *

80	Colombian	0/6	19/6	0/5	*0.001 *	NS	NS
150	Colombian	0/6	14/6	6/5	*0.001 *	*0.002 *	NS
240	Colombian	0/6	39/6	10/5	*0.001 *	*0.002 *	NS

ECG record of mice genetically vaccinated and challenged as described in the legend of Figure 5(a). Values represent the number of episodes (sinus arrhythmia or atrioventricular block) recorded during one minute per total of mice analyzed and were compared by Fisher exact probability test.

**Table 2:** Summary of the ECG records.

Days after challenge	Strain	Sinus arrhythmia mouse groups			*P* values		
		G1	G2	G3	G1 × G2	G1 × G3	G2 × G3
90	Brazil	0/6	10/6	0/6	*0.001 *	NS	*0.012 *
120	Brazil	0/6	10/6	1/6	*0.001 *	NS	*0.044 *
150	Brazil	0/6	11/6	5/6	*0.001 *	*0.007 *	NS
180	Brazil	0/6	4/6	4/6	0.03	0.03	NS

90	Colombian	0/6	3/6	1/5	NS	NS	NS
120	Colombian	0/6	3/6	0/5	NS	NS	NS
150	Colombian	0/6	12/6	0/5	*0.001 *	NS	*0.013 *
180	Colombian	0/6	16/6	1/5	*0.001 *	NS	*0.022 *

Days after challenge	Strain	Atrioventricular block mouse groups			*P* values		
		G1	G2	G3	G1 × G2	G1 × G3	G2 × G3

90	Brazil	0/6	36/6	46/6	*0.001 *	*0.001 *	NS
120	Brazil	0/6	19/6	22/6	*0.001 *	*0.001 *	NS
150	Brazil	0/6	5/6	6/6	*0.007 *	*0.001 *	NS
180	Brazil	0/6	5/6	3/6	*0.007 *	NS	NS

90	Colombian	0/6	20/6	30/5	*0.001 *	*0.001 *	NS
120	Colombian	0/6	27/6	15/5	*0.001 *	*0.001 *	NS
150	Colombian	0/6	6/6	2/5	*0.001 *	NS	NS
180	Colombian	0/6	10/6	10/5	*0.001 *	*0.001 *	NS

ECG record of mice challenged and treated as described in the legend of Figure 6(a). Values represent the number of episodes (sinus arrhythmia or atrioventricular block) recorded during one minute per total of mice analyzed and were compared by Fisher exact probability test.
